# Disturbances of Shared Intentionality in Schizophrenia and Autism

**DOI:** 10.3389/fpsyt.2020.570597

**Published:** 2021-02-10

**Authors:** Alessandro Salice, Mads Gram Henriksen

**Affiliations:** ^1^Department of Philosophy, University College Cork, Cork, Ireland; ^2^Center for Subjectivity Research, University of Copenhagen, Copenhagen, Denmark; ^3^Department of Communication, Center for Subjectivity Research, University of Copenhagen & Mental Health Center Amager/Glostrup, Copenhagen, Denmark

**Keywords:** anomalous self-experience, autism spectrum disorder, group identification, mind reading, perspective-taking, schizophrenia, self-disorders, shared intentionality

## Abstract

Schizophrenia and autism are today considered complex spectrum disorders characterized by difficulties in social behavior. Drawing on recent advances in collective or shared intentionality studies, we present a novel theoretical approach to these social difficulties by exploring them from the angle of shared intentionality. We begin by describing two forms of shared intentionality: joint intentionality and we-intentionality. Joint intentionality crucially relies on the agents' mentalizing abilities such as mind reading and the ability to factor in (or “to be moved” by) their partner's intentions in deliberation and action planning. By contrast, we-intentionality relies on the agents' capacity to understand themselves as group members and to adopt the group's perspective. In schizophrenia spectrum disorders, we propose that joint intentionality remains unaffected, but we-intentionality may be impaired. In severe autism spectrum disorder (i.e., infantile autism), we propose that both forms of shared intentionality are impaired. We suggest that the source of the problems affecting we-intentionality in schizophrenia spectrum disorders lies primarily in trait-like, anomalous self-experiences. In severe autism spectrum disorder, we suggest that problems with mind reading, the ability to “be moved” by others' intentions, and with the capacity for perspective-taking impede both forms of shared intentionality.

## Introduction

In the last decades, collective or shared intentionality has attracted rapidly growing attention in many research communities. Shared intentionality can be described as the power of the mind to share mental states like emotions, intentions, and beliefs with others [see (1)]. Philosophers and empirical researchers have argued that this capacity is of paramount importance for characteristically human forms of social life, because it appears to underlie key social phenomena, including communication (2), cooperation (3), group and corporate agency (4), the constitution of institutional facts (5), human moral psychology (6), and collective responsibility (7). By uncovering how pervasive shared intentionality is in human life, this wealth of insights also supports the prediction that disturbances of this capacity will reflect noticeable changes in human sociality.

This prediction delivers the background motivation of this paper, whose principal aim is to shed new light on the nature of aberrant social behavior in schizophrenia spectrum disorders (i.e., schizophrenia and schizotypal disorder, hereafter “SSD”) and autism spectrum disorder (“ASD”). The social behavior in these disorders has been a subject of intense research for decades [see (8, 9)], typically associating such behavior with various forms of neurocognitive and social cognitive deficits. Previous studies have generally not explored aberrant social behavior from the perspective of shared intentionality except for some sporadic contributions [these include (10–13)]^1^. We suggest that recent advances in studies on shared intentionality may offer a new framework for understanding the characteristic impairments of sociality in SSD and ASD and for illuminating crucial differences in social impairments in these diagnostic groups. Furthermore, appreciating the specific nature of these impairments in the two disorders may enable us to better comprehend the features of shared intentionality that are required for it to function unproblematically.

The paper is organized as follows. In Joint and We-Intentionality and Their Core Features section, we develop a conceptual framework for thinking about shared intentionality. We claim that shared intentionality comes in at least two forms, which we label “joint intentionality” and “we-intentionality,” and that they have different core features and psychological preconditions. In short, joint intentionality requires mentalizing abilities such as mind reading and the ability to be “moved” by the intention of another agent (Joint Intentionality section). By contrast, we-intentionality crucially hinges on group identification, which is the capacity to acquire a self-understanding as group member and to adopt the group's perspective (We-Intentionality section).

In Schizophrenia Spectrum Disorders section, we advance theses partially already defended in previous work (10), which specifically concerned sociality in schizophrenia^2^. We present reasons for thinking that, of the two forms of shared intentionality identified in Joint and We-Intentionality and Their Core Features section, only one of them seems to be impaired in SSD. Whereas, joint intentionality does not appear to be affected in SSD, we suggest that we-intentionality can, in fact, be impaired in SSD (Social Behavior in Schizophrenia Spectrum Disorders section). We propose that the difficulties of we-intentionality are linked to the presence of non-psychotic, anomalous self-experiences (or “self-disorders”), which contemporary research documents hyper-aggregate in SSD but not in other mental disorders [see (14)], and which can be considered to be trait-like features of SSD, antedating psychosis and persisting after remission from a frank psychotic episode (15, 16). We propose that the anomalous self-experiences may hamper the process of group identification, thereby potentially impairing the formation and maintenance of we-intentionality (Frailty of We-Intentionality in Schizophrenia Spectrum Disorders section).

In Autism Spectrum Disorder section, we look into ASD by zooming in on the severe end of the spectrum [what in *International Classification of Diseases*, Tenth Revision (ICD-10) is termed “infantile autism”]. It is therefore important to highlight already now that our account, by exclusively focusing on the severe end of the spectrum, deliberately leaves aside milder cases of autism (i.e., Asperger's syndrome) and so-called high-functioning autism. After some general considerations concerning sociality in severe ASD (Social Behavior in Severe Autism Spectrum Disorder section), we present the hypothesis that, in severe ASD, both forms of shared intentionality are disrupted (Joint Intentionality in Severe Autism Spectrum Disorder section). We argue that the problems with mentalizing abilities and the capacity for perspective-taking, which the current literature has already acknowledged as a qualifying trait of severe ASD, have negative repercussions for initiating interactions based on both joint intentionality and we-intentionality (We-Intentionality in Severe Autism Spectrum Disorder section)^3^.

Before approaching the notion of shared intentionality, one last remark is in order: part of the motivation for this project stems from a general absence of empirical research around shared intentionality in SSD and severe ASD. Against this backdrop, the following sections try to break new ground by offering a novel theoretical or conceptual account of the disturbances of shared intentionality in the two syndromes. Evidently, there is no available experimental design to test our account or empirical evidence to validate or falsify it. Yet we draw on both classic and contemporary research to get to the psychopathological core of the two syndromes and develop our account. The hope is that this paper may contribute to open a line of research on shared intentionality in psychopathology in which the basic hypotheses of the presented account may be tested.

## Joint and We-Intentionality and Their Core Features

Philosophy of mind usually distinguishes among a general and a specific meaning of the term “intentionality.” When used in the general sense, intentionality is a property of mental states: a mental state qualifies as intentional if it is about an object or a fact in the world (17). On this understanding, shared intentionality refers to the power to share mental states that are intentional. Accordingly, investigating the ways in which, say, perceptions, emotions, or beliefs are shared among several individuals is part and parcel of the investigation into this power^4^.

When used in the specific sense, intentionality is a property of actions: an action is intentional if it is performed upon a conative state like an intention (20). On this second understanding of “intentionality,” shared intentionality narrowly refers to the power of sharing states of a conative kind and, especially, intentions. Although an exhaustive assessment of impaired sociality in SSD and ASD demands investigations of how sharing of cognitive or emotive states is affected in the two conditions, the present paper will exclusively focus on the “specific” sense of “shared intentionality” as the capacity to share conative attitudes and, more specifically, intentions. However, it should be noted already now that an explanation of this capacity will not exclusively appeal to conative states and attitudes. Rather, as it will turn out, sharing intentions relies on a host of psychological preconditions that also include cognitive and emotional states.

To intuitively corroborate what is meant by the expression “shared intention,” imagine two individuals walking down the street (21). For our purposes, this scenario can play out in two different ways. First, the two individuals may be performing *distinct* actions in strategic equilibrium. Here, each individual monitors what the other is doing in order to avoid disruption in one's course of action, e.g., accidentally stepping on the other's foot. Second, the individuals may be performing an action *together*. It has been convincingly argued [e.g., by (21–24)] that what distinguishes the first scenario from the second is the fact that the individuals, in the latter, walk together because they *have jointly decided to walk together* or, to put this another way, because they *share the intention of walking together*. A large part of the current debate concerns what, exactly, it means for several individuals to “share” intentions. Recently, one view is gaining significant traction in the literature. According to this view, “sharing” does not point to just one thing, as it were; rather, there are different ways in which mental states like intentions can be shared [authors endorsing this idea include (3, 25, 26), among others]. In the following, we develop a conceptual framework that aims at capturing two different ways of sharing intentions [see (10, 27, 28)].

### Joint Intentionality

We call the first form of sharing “joint intentionality.” In joint intentionality, agents pursue individual goals that happen to overlap, where a goal is the state of affairs that an agent is committed to bring about. For example, imagine that I intend to write a paper and you intend to write a paper as well. In this case, our individual goals (to write a paper) can be said to overlap [at least to a certain extent, see (29)]. Suppose that I become aware of your intention and you of mine: assuming some favorable circumstances (we esteem each other, or we have complementary expertise, etc., the details are irrelevant for our purposes), this may motivate me to form the intention to write the paper together with you on condition that you, too, intend to do so. So I decide to write the paper together with you, “partly because” you, too, have the intention of writing a paper with me (24). But also, I form the intention in “accordance with” yours, where accordance is required to exclude cases of exploitation or coercion, in which I use you as a mere social tool or against your own interests (24). Thus, we propose the following two psychological preconditions for intentions to be “shared” in a way leading to a jointly intentional activity: (1) I am aware that you have a mental state, which qualifies as an intention (“mind reading”), and (2) this intention of yours figures in my pool of motivations in a particular way; i.e., for our intentions to lead to intentional joint action, your intention must “move” me in the sense that I factor in your intention in my deliberation and action planning by forming my participatory intention “in accordance” with, and “partly because” of, yours. When individual intentions—i.e., intentions that are held from the agents' individual perspectives and are the endpoint of a deliberative process aimed at solving a practical problem that each of the individual agents is confronted with—are formed this in specific way, they may be called “participatory intentions.” To put this differently, two or more individuals engage in joint intentionality when each of them forms participatory intentions.

Once participatory intentions are in place, a further requirement for them to lead to intentional joint action is shared deliberation about the plan and the distribution of labor. To elaborate on the example, either concomitant or expected deliberation about—and subsequent agreement on—which part of the paper will be written by whom is part and parcel of what it means for you and me to decide to write a paper together (24). This implies that the interactants put themselves under the pressure of assigning roles and statuses based on their specific features, expertise, and capabilities (30, 31). Of course, such pressure may be minimal (or practically inexistent) in very simple interactions where the course of action is evident to the agents, but it can also peak in case of complex interactions where the agents' stakes are very high. Importantly, the rules based on which such roles and statuses are assigned (as well as the agents' intentions that initiate the joint action) will typically be formulated in an explicit way, which secures common knowledge about them among participants. Usually, common knowledge is described as a set of recursive beliefs that range over others' (recursive) beliefs. On this view, a proposition *p* is common knowledge in a population *n*, if everybody in *n* knows (and, thus, believes) *p*, everybody in *n* knows (and, thus, believes) that everybody in *n* knows (and, thus, believes) *p*, etc.

To be sure, it is very much debated in the literature whether common knowledge is indeed required by joint intentionality and how the notion ought to be understood (32–34). Yet many prominent accounts concur that common knowledge indeed is an important feature of joint intentionality, which is required to make all parties informed about the fulfillment of the above-described psychological preconditions [see (23, 25, 35, 36)].

Another characteristic of joint intentionality should not go unnoticed: the intentions had by the individuals come in the “I-form” [or “I-mode,” see (25)]. In other words, individuals form and maintain intentions from their own *individual* perspective^5^. Basically, this means that an interaction steered by joint intentionality is initiated by intentions, which the agents form on the basis of *individual* reasons and motives (in the example at stake: your and my individual intention of writing a paper) and which they entertain from their *individual* perspectives. Another way of putting this is that, in joint intentionality, agents have the unilateral power to break apart the shared intention by a simple change of mind—if an alternative emerges, which is more appealing to the individual, this individual is free to give up on his or her intention and pursue another option [(35), p. 79]. This is also why agents operating on the basis of joint intentionality often monitor each other with circumspection—one agent is motivated to invest efforts in the joint activity only as long as, and to the extent to which, the other agent, too, invests resources in the activity (thereby signaling that they remain committed to their individual goal) and vice versa.

### We-Intentionality

Things look differently if one turns to “we-intentionality.” Here, individuals occupy mental states in the “we-form” [or “we-mode,” see (25)], which are poised to be reported by employing the first-personal plural pronoun (“we intend …”). For example, imagine that some friends decide to cook dinner together by each of them forming an intention of the form “we intend to cook dinner.” In this case, the goal is *not* shared *distributively* as in joint intentionality, where the individual goals happen to overlap. Rather, in we-intentionality, the goal is understood as a group's goal, which all group members, *collectively*, are committed to bring about. Differently put, each individual forms a we-intention that aims at a goal, which is framed as collective or as a group's goal and the achievement of which the individuals are committed to. Importantly, because of this commitment to the achievement of the group's goal, agents do not have unilateral power to dissolve their we-intentions—if one individual considers giving up on the joint action, some form of permission for doing so should be sought in the other parties (35)^6^.

Engaging in we-intentionality appears to require at least two elements. The first is that individuals must be able to answer the question “Who am I?” by saying: “I am one of us” (39). More specifically, they must be able to understand themselves as group members. This self-understanding as a group member elicits a subjective sense of group membership (39), belongingness (40), or we-ness (41), which transforms the agent's self-experience into a self-experience as a group member, thereby delivering the motivation to form and entertain we-intentions. In other words, insofar as agents see themselves as group members, they are motivated to act as such^7^.

The second requirement of we-intentionality is that agents must be able to answer the question “What should we do?” by referring to the group's goals or preferences (42): “we intend to φ.” This presupposes the capacity to take the group's perspective or the “we-perspective” (43–45) and, thereby, to frame the world from the perspective of one's group^8^. Adopting this perspective also provides the agents with “group nous” (48) or “group ethos” (25), i.e., with practical knowledge on how to plan their conduct and to efficiently adapt it to the group's goal.

We subsume the process of acquiring a self-understanding as group member and the capacity to adopt the group's perspective under the umbrella term of “group identification” [(27); see also (49, 50)]. We will elaborate on the issue of group identification in the next sections, but it should be emphasized already now that group identification may happen even in the absence of previous interaction among the agents. Given certain conditions, to which we come back especially in Frailty of We-Intentionality in Schizophrenia Spectrum Disorders section, total strangers may group identify and, thereby, acquire the disposition to collaborate. This may suggest that the difference between joint and we-intentionality does not hinge on pre-existing relations among the involved individuals—both forms of shared intentionality may build upon previously existing relations, but both could also be activated even in the absence of those relations^9^.

There are important differences between interactions based on joint intentionality and interactions based on we-intentionality. First, when steered by we-intentionality, the whole interaction assumes a spontaneous character—the other does not need to be monitored constantly but is *trusted* to deliver the contribution to the joint activity because the other, as oneself, is framed as an in-group member [see (52)]. This is a form of trust described in social psychology under the label of “depersonalized trust,” where it designates a trust that is “extended to any member of the ingroup whether personally related or not” [(53), p. 433] just in virtue of the fact that the other has been framed as an in-group member. In addition, in these interactions, the agents are not under the relentless pressure of deliberating about the plans: things can be done the way “we” do, by substantially relying on shared *common sense*^10^. Obviously, this does not imply that agents will not scrutinize, revise, or reassess the group's plan at any point in time where this may be required. Shared deliberation about means and distribution of labor is and remains in the service of shared agency, but the pressure on the agents to engage in action planning is arguably more limited than in joint intentionality scenarios.

The discussion of these two forms of sharing is not meant to be exhaustive and leaves open several important questions such as whether these two forms of shared intentions are distinct in kind (or just in degree of, e.g., cognitive complexity), which form is ontogenetically and phylogenetically more primitive^11^, whether there are yet other forms of shared intentions, and whether sharing of beliefs and emotions operates in the same way as sharing of intentions. These questions already show that we are not proposing a “one-fit-all” account of shared intentionality. However, we do suggest that this conceptualization of shared intentionality and especially the description of the main features and psychological preconditions of joint and we-intentionality (as summarized in Tables 1, 2) may be a valuable theoretical framework for understanding the impairment of sociality in SSD and severe ASD.

**Table 1:** Core features of joint and we-intentionality.

	**Goals**	**Perspective**	**Interpersonal stance**
**Joint intentionality**	Individual	Individual perspective	Circumspection
**We-intentionality**	Collective	Group's perspective	Trust

**Table 2:** Psychological preconditions of joint and we-intentionality.

	**Psychological preconditions**	
**Joint intentionality**	Mentalizing abilities	
	Mind reading	“Being moved” by the other's intention
**We-intentionality**	Group identification	
	Self-transformation	Adoption of the group's perspective

Before approaching how shared intentionality is disrupted in SSD and ASD, it is important to add a few further details to this picture to avoid potential misunderstandings. *First*, talking of we-intentionality in the context of this paper is talking of intentions had by individual agents, who have group identified, and where group identification is a psychological process that elicits as *subjective* sense of group memberships (i.e., one frames oneself as an in-group member). While one can speculate that an *objective* sense of group memberships (i.e., the social fact that an individual belongs to a certain group) must be related to a subjective sense of group memberships, our paper is entirely focused on those joint actions that are enabled by a subjective sense of group membership. *Second*, our paper takes shared agency in informal and small-scale groups as its main explanandum and remains largely silent on agency in large and institutionalized groups, and on their relation to shared intentionality. However, it should be noted that we do not see any straightforward relation between informal, small-scale groups and joint intentionality, on the one hand, or between large, institutionalized groups and we-intentionality, on the other. Just as we-intentionality can be activated in dyadic joint action, so can joint intentionality be activated in large-scale corporate agency. So, for instance, it could be that an individual agent's goal and a group's goal overlap—in this case, the individual agent may form a participatory intention with another agent in the sense of joint intentionality (it just so happens that the other agent is a group agent). *Third*, because factors like trust, collective goals, and the group's perspective are inherent in we-intentionality, and because they enable, regiment, and sustain joint activities, we-intentionality can steer activities that do not require plans, rules, structure, norms, etc. (which, however, is not to say that we-intentionality cannot also steer activities that are planned, structured, normed, etc.). By contrast, precisely because joint-intentionality lacks those factors, it is conducive to activities that require plans, rules, and structure.

## Schizophrenia Spectrum Disorders

The contemporary diagnostic manuals, i.e., ICD-10 (60) and *Diagnostic and Statistical Manual of Mental Disorders*, Fifth Edition (DSM-5) (61), define schizophrenia as a psychotic disorder, characterized by delusions, hallucinations, catatonia, severe formal thought disorders (e.g., incoherence), and negative symptoms (e.g., decreased emotional expressivity). Schizotypal disorder is defined slightly differently in the two manuals: ICD-10 lists it immediately after schizophrenia [(60), p. 95], whereas DSM-5 lists it among the personality disorders [(61), p. 655]. However, there is general agreement that schizotypal disorder is a part of the schizophrenia spectrum [(61), p. 90].

These manuals also acknowledge interpersonal difficulties that may accompany schizophrenia, e.g., impoverished interpersonal relations [(61), p. 99] and social withdrawal or lowered social performance as a result of negative symptoms [(60), p. 88]. DSM-5 describes schizotypal disorder as a “pervasive pattern of social and interpersonal deficits marked by acute discomfort with, and reduced capacity for, close relationships” [(61), p. 655]. In addition, “lack of close friends or confidants other than first-degree relatives” forms a diagnostic criterion; ICD-10 lists “poor rapport with others and a tendency to socially withdraw” as a criterion. Classical accounts of SSD [e.g., (57, 62–64)] emphasize that interpersonal difficulties are not some additional or marginal aspect, e.g., mere sequela of psychosis, paranoid ideation, or suspiciousness, but an integral, often persistent part of SSD.

In the following, when we explore aberrant social behavior in schizophrenia, we will therefore not zoom in on abnormalities of behavior that primarily co-occur with psychotic or near-psychotic episodes [e.g., walking naked in the streets, mutism, or the so-called “crazy actions”; (65–72)], which in themselves reflect a dislocation from the shared-social world. Rather, we will key in on more pervasive and persistent interpersonal difficulties that regularly are found in SSD, and which classical psychopathologists associated with the Bleulerian concept of *schizophrenic autism* [see (62), p. 63ff], which should not be conflated with the notion of autism that arose from the work of Kanner (73) and Asperger (74), and which has formed the basis of the concept of ASD (see Social Behavior in Severe Autism Spectrum Disorder section).

### Social Behavior in Schizophrenia Spectrum Disorders

When approaching the topic of sociality in schizophrenia, one is likely to encounter the following puzzle. On the one hand, patients with SSD often report continuous difficulties in establishing and maintaining social relations with others, and frequently these difficulties are a source of loneliness and isolation. On the other hand, patients may simultaneously report that they really enjoy and often participate in various forms of social interactions. What is puzzling is of course not that patients participate in all kinds of social interactions, despite the difficulties they may experience, but that some of these social interactions apparently are experienced as easy and enjoyable, whereas other interactions are experienced as almost intolerable. Yet it remains unclear what constitutes this significant difference. How can we explain this puzzle?

In previous work (10), we have described, based on anecdotal clinical experience over many years, that social activities such as karate, ballet, board games, live action role-playing, and massively multiplayer online game (MMOG) often seem to be experienced as quite unproblematic. By contrast, other activities such as spontaneous or informal social interactions or establishing and maintaining close friendships over an extended period of time often are experienced as difficult. A few examples from patients with SSD may help illustrate our points.

One patient, who regularly isolated himself for months, participated in a weeklong live role-playing game with many people he had never met before. He said, “There I could be myself in a way I haven't been able to since high school. When I play, I am ‘in character' in a world, where B necessarily follows from A. It's a universe that you control yourself and unlike the real world, there's always a reason for what's happening” (75). A recovered patient, now working as a teacher, felt most interpersonal exchange, apart from that she had with her intimates, deeply uncomfortable. However, her professional life provided her with an important exception. She said, “I was surprised at how well it went (…) I think it's because I have a foundation in talking about professional stuff and the students don't expect that you small-talk a whole lot with them (…) There I'm playing a part, I have a certain role, I kind of have a function” (75). Another patient, a nursing student, describes how she avoids spending time with her colleagues during breaks, because the small talk makes her uncomfortable. Instead, she prefers to be around patients. She said, “I think I might have a bit more energy when I'm wearing my uniform (…) Then I have a part to play. Then I have to be a nursing student and I know what to say and what not to say (…) It's kind of like there are more written rules on how to behave, and that's more difficult when you're just being yourself.” In her spare time, she reports being involved in eight groups of friends that all are organized around discrete activities. She said, “Compared with many of my friends who just get together without doing anything, I'm like (…) there needs to be some kind of point in meeting up or a kind of purpose.” For her, one such purpose was badminton—“Then there's badminton, and it's from seven to nine, and that's it, then it's over” (76).

We have suggested that one answer to the question of the puzzling social behavior in SSD may be that some of these activities predominantly correlate with joint intentionality, whereas others predominantly correlate with we-intentionality^12^. In our view, the hypothesis that best coheres with the observations about social behavior is that patients with SSD regularly may find interaction based on we-intentionality difficult, whereas they typically do not encounter problems with joint intentionality. As we have argued in Joint Intentionality section, it is usual for interactions steered by joint intentionality to have quite neatly defined roles, to be structured (some of these interactions are ritualized), and to rely on a set of explicitly formulated rules. These features of social interaction based on joint intentionality are vividly described in the examples above. We have argued that these features evoke a sort of tranquilizing effect insofar as they contribute to make the activities and the social context in which they occur predictable, reliable, and essentially safe. Differently put, the uneasiness, confusion, and pervasive anxiety that many patients with SSD may experience in social situations are counteracted or balanced by these features as they enable participants to know *what* to do, *how* to do it, and *when* to do it [(10), p. 160]. Further studies into the social life world of patients with schizophrenia indicate that patients may, in fact, adopt joint intentionality in social contexts and relationships, where one perhaps would expect to find we-intentionality (75, 76). For example, patients may actively employ various “compensatory strategies” to navigate the social world, e.g., imposing a spatiotemporal structure on social interactions (that typically would not necessarily possess such a structure) and seeking out or preferring activities marked by a clear distribution of social roles and rules (75). In our experience, patients do not regard such compensatory strategies as constraints ideally to be overcome but rather as a structure on which their involvement with the social world hinges (75). In other words, such compensatory strategies, which, at least to some extent, exploit the resources of joint intentionality, seem to help patients live a social life, stabilize the conditions, and promote recovery [see (77)].

At this stage, it is important to note that the observations about the predilections of ritualized and structured joint activities in SSD could be claimed to be compatible with the possibility that the patients do activate we-intentionality when interacting. As we have suggested above, the correlation between joint intentionality and a structured form of agency is merely stronger in joint intentionality, but this does not exclude the possibility of rigidly structured interactions, which are steered by we-intentionality. So what does support the hypothesis that, in SSD, we-intentionality (but not joint intentionality) is disrupted?

The prevailing view in the literature is that impaired social functioning in schizophrenia is caused by social cognitive or neurocognitive deficits, which have been found to explain 20–60% of the variance of social functional outcome in schizophrenia (78). Thus, a considerable proportion of the variance remains unexplained, motivating a continued search for other relevant factors or mediators. Our suggestion is that the fairly specific psychopathological profile of SSD, viz., the aggregation of anomalous self-experiences in SSD, is a key source of these patients' difficulties in the interpersonal domain and, more specifically, that the aggregation of anomalous self-experiences exerts friction on the process of group identification, which, as described above, is a psychological precondition for activating and maintaining we-intentionality. In order to better explain our claim, we will, therefore, explore in some detail the notions of group identification and anomalous self-experience. We start with group identification and then discuss how certain anomalous self-experiences may destabilize this mental process.

In We-Intentionality section, we have introduced “group identification” as an umbrella term for two different processes. On the one hand, transformation in self-experience enables the formation of we-intentions. On the other, the adoption of the group's perspective, understood as a specific process of perspective-taking, delivers information to the agent about the group's preference or goal by instructing him or her on how to act based on the expectations and predictions of how the group will act. We will postpone a more thorough discussion of this second aspect of group identification till we turn to severe ASD (see We-Intentionality in Severe Autism Spectrum Disorder section). For now, we focus on the transformation in self-experience.

Such transformation of self-experience can be triggered quite easily as experiments conducted since the early 70s on the so-called minimal group paradigm illustrate [see (79, 80)]. This branch of research also shows that several conditions need to be fulfilled for a self-conception as “group member” to be acquired. What then are these conditions? First, the individual should be aware of what has been labeled “group cues” (49), which include having common interests, sharing a common fate, facing a competing group, and using we-language [(43); we return to these cues in We-Intentionality in Severe Autism Spectrum Disorder section, where we shall discuss a particularly important cue, namely, joint attention].

For now, it suffices to state that when a subject perceives these group cues, they can trigger two interrelated consequences. The first is “self-categorization,” which conduces subjects to see themselves as saliently similar to the others (those who, say, have the same preferences, exemplify the same properties, or are in the same life condition, etc.). The second is what social psychologists call “depersonalization,” which is described as “a shift toward the perception of self as an interchangeable exemplar of some social category and away from the perception of self as a unique person defined by individual differences from others” [(39), p. 50]. Because the term “depersonalization” also denotes both a psychiatric symptom and a disorder [(61), p. 302ff], we will refrain from using this term and instead use the term “de-individuation” to avoid potential confusions.

From the perspective of social psychology research, the ultimate effect of self-categorization and de-individuation is the acquisition of a self-understanding as group member or a “social self” (81). By conceiving of myself as member of a group (to which you, too, belong), I am moved to behave as a group member^13^.

### Frailty of We-Intentionality in Schizophrenia Spectrum Disorders

Let us now explore how various anomalous self-experiences may counteract group identification and, more specifically, the interrelated process of self-categorization and de-individuation.

It is important to keep in mind that anomalous self-experiences are not discrete, atomic-like symptoms but mutually implicative aspects of the psychopathological *Gestalt* of the schizophrenia spectrum (85, 86). Empirical studies have documented that, on average, patients with SSD have ~20 anomalous self-experiences, and this is significantly more than what has been found in all other mental disorders (66–72, 87, 88). Overall, the empirical studies on anomalous self-experiences seem to support the idea that the basic disturbance in schizophrenia spectrum disorders is a disorder of ipseity or minimal self (14, 89–91)].

In the following, when we address a few singular anomalous self-experiences and discuss how they individually may impede self-categorization and de-individuation, this is done strictly for expository purposes. Other anomalous self-experiences may impede these processes as well (e.g., thought pressure, ambivalence, inability to distinguish modalities of intentionality, diminished sense of being present in the world, and quasi-solipsistic experiences), but they will not be explored here. Furthermore, we are not ruling out the roles that deficits in theory of mind, neurocognition, or social cognition may have on group identification, and we have no reason to believe that such roles should somehow be inconsistent with the role that we here ascribe to anomalous self-experiences—e.g., one study found that patients with first-episode schizophrenia under-interpreted social cues and over-interpreted non-social cues (92). There is a long tradition of research on theory of mind deficits in schizophrenia (93). While such deficits perhaps also may exert friction on the process of group identification and thus we-intentionality, these deficits do generally not appear to be so severe that they hamper the psychological preconditions for joint intentionality in SSD (we discuss this issue in the end of Joint Intentionality in Severe Autism Spectrum Disorder section).

In the following, we first summarize a few anomalous self-experiences and the process they may impact before subsequently exploring these issues in further detail. In our view, self-categorization by which subjects perceive themselves as saliently similar to others is often destabilized by a feeling of being different from others (*Anderssein*) and problems involving common sense problems.

First, “Anderssein” refers to enduring and pervasive feelings, which usually have been present since childhood or early adolescence, of being different from others or simply “wrong” as some patients put it [(14), p. 253]. In short, it is a profound feeling of inner and existential alienation. Nagai has aptly stressed the difficulty in understanding this feeling of being different in schizophrenia [(94), p. 497]. Usually, when we speak of differences, we presuppose a shared domain in which such differences occur and are measurable against each other. But in the case of “Anderssein” in schizophrenia, Nagai suggests that there is no such shared domain and that we are instead faced with a non-objectifying, contentless feeling of difference [(94), p. 497f]. In other words, we are dealing with a global feeling of difference that often resists verbalization and precedes thematization, i.e., finding out “what” is different. Nonetheless, patients often search for and find some explanation for their pervasive feelings of difference (e.g., “it's my low self-esteem” or “I am an introvert”), but when explored in depth, such explanations usually do not fully exhaust their profound feeling of difference, which often appears to be rooted in a much deeper sense of “being ontologically different” [(14), p. 253]. While many patients struggle to convey the quality of this feeling of difference, others are able to express it in quite illustrative ways. For instance, one patient said, “I looked just like every other child, but inside I was different. It is as if I am another creature that somehow ended up inside a human body” [(95), p. 436]. Another patient said, “I've always felt as if others could almost smell that I was different. They could simply feel that I was a different animal in the herd. I always felt like a giraffe among rhinos” (75). Yet another patient described how he already from childhood felt lonely, insecure, and different from others. At one point, he asked his mother if he was robot because, as he said, “I felt like I was a machine … if one could remove the face, then I thought there would be a machine inside or perhaps some other creature” [(96), p. 180]. In our view, such profound feelings of *ontological dissimilarity* may impede recognition of more *mundane similarities* (e.g., similar taste in music) or make such similarities appear superficial or arbitrary, thereby impeding self-categorization and, thus, group identification [(10), p. 162f].

Second, feelings of being different from others often go hand in hand with various problems of common sense [(57, 58), p. 307f]. The heart of common sense problems appears to be a failing of automatic, pre-reflective attunement in the person's self-, other-, and world-relation [(14), p. 253]. Common sense problems often manifest as an inability to simply take for granted what others consider obvious or matter of fact. One patient offers a vivid description of how she experienced these issues—she said,

I have always struggled to understand why people didn't take life more seriously. I mean, “How can you just walk around, be named ‘Angie,' buy butter, and take riding lessons?” Every morning, when I wake up, I realize like for the first time that this is the real reality, that we are all going to die, that we don't know why we are here, that nothing makes sense … This is one of the reasons why I feel different from others. They walk around and talk on their phone, plan what they want to do … It puzzles me that I haven't gotten used to it. Everyday I realize that the sky is just above us, right … infinity is so near, we don't know why we are here, and we will all die … It hurts me that it is so easy and natural for the rest of the world. They don't even think about it [(95), p. 267].

Another patient reported that she often pondered questions such as “why a table is called a table or why humans only have two arms instead of four or why the arms aren't placed lower to the ground, which would make it easier to pick up things” [(96), p. 180]. As Stanghellini (97) has argued, the crisis of common sense in schizophrenia does not only concern subject–object relations but crucially also the subject–subject attunement. This was also the case for this particular patient—she said, “I speculate a lot on why people do what they do? I often don't get it” [(96), p. 180]. As the examples indicate, common sense problems are typically associated with tendencies to hyper-reflect about oneself, others, or objects in the environment, often in an attempt to decode their meaning. In our view, common sense problems and hyper-reflection may impede recognition of group cues, e.g., by disallowing relevant properties to stand out as salient in social contexts. The prediction that these considerations justify is that, again, this particular anomalous self-experience may impede self-categorization.

Next, we suggest that the process of de-individuation by which subjects deemphasize individual differences in favor of properties that are shared with others is destabilized by experiences of hyper-reflection/self-monitoring and transitivism.

First, as implied above, the objects of hyper-reflection may not only be others or objects in the environment but also aspects of oneself. For example, one patient reported that his central problem concerned difficulties with engaging and remaining in relationships with others [(98), p. 206–208]. Starting a conversation was very difficult for him, and, at one point in his life, he stopped communicating with others altogether. He feels that “starting a conversation with someone implies taking over responsibility for the relationship, especially for the next step. Because he feels paralyzed at the same time, he doesn't dare to even start a conversation. The scenarios, which are constructed in his head before any relationship even takes place, completely block him” [(98), p. 207]. Similarly, other patients report that they, before starting a conversation with someone, prepare themselves minutely by imagining and playing out all possible routes the conversation may take (99). In other cases, hyper-reflection may lead to excessive forms of self-monitoring that are operative alongside the subject's engagement with others. For example, one patient reported how this made social interactions difficult for her:

I always feel that it is like enormously feigned when I have some social interaction. It feels false, like I can't react naturally or sincerely like everyone else … I have the experience that there are two of me: the one that interacts with someone and then there is the real me, who sits there behind. For example, “I sense that the one I'm talking to finds my statement a little transgressive, so I add a little humor here to establish an ironic distance. That may perhaps … yes, that worked well …” And I do it, like, simultaneously. I don't feel present at all [(95), p. 267].

In our view, the self-involvement that is at stake in such experiences of hyper-reflection and self-monitoring may not only render fluid, spontaneous interactions with others difficult but also impede the subject from de-emphasizing individual differences as required in de-individuation.

Second, transitivism (sometimes also referred to as “demarcation problems” or “problems with ego-boundaries”) denotes a group of experiences that are characterized by permeability of the me/not-me boundary. According to Schneider, most of the first-rank symptoms of schizophrenia (e.g., thought insertion, withdrawal or broadcasting, and other passivity phenomena) fundamentally involve transitivism—a “loss of the very contours of the self” [(100), p. 134; see also (101)]. Experiences of transitivism are frequently reported in SSD. For example, patients may describe experiences of being somehow “mixed up” with another person, not knowing what side of the mirror they are on, or more pervasive experiences of being “too open” or “without any barriers” (102). One patient reported being very anxious among others, whom she felt “can see through me and see all the bad things I have done in my life” [(96), p. 180]. Parnas and Handest (103) offer another illustrative vignette:

A young man was frequently confused in a conversation, being unable to distinguish between himself and his interlocutor. He tended to lose the sense of whose thoughts originated in whom, and felt “as if” his interlocutor somehow “invaded him,” an experience that shattered his identity and was intensely anxiety provoking. When walking on the street, he scrupulously avoided glancing at his mirror image in the windowpanes of the shops, because he felt uncertain on which side he actually was. He used to wear a wide and tight belt in order to feel “more whole and demarcated” [(103), p. 130].

In our view, experiences of transitivism, which usually are experienced as very disturbing, may also affect the process of de-individuation. It seems at least possible that patients who already feel vulnerably transparent and too open may want to resist de-emphasizing individual differences.

One could question whether the non-psychotic anomalous self-experience, which we have described here, in and of themselves also could impact joint intentionality. In our view, this is not the case. Although patients often feel different from others, joint intentionality, unlike we-intentionality, does not hinge on group identification. Patients also often report problems with common sense (e.g., a failing grasp of the implicit rules of social interaction), regularly accompanied by hyper-reflection. However, such confusion in social interaction is largely bypassed in joint intentionally, which typically has a well-defined goal and rely on explicitly formulated rules and roles, securing common knowledge among the participants. With regard to transitivism, it is important to emphasize that although patients, in certain situations, may feel “as if” others, merely by looking at them, can know what they are thinking, they actually know that this is not the case (as implied in the conditional “as if”). In other words, the ego-boundaries, though sometimes felt as frail or permeable, are not dissolved. Thus, it does not follow that the patients' capacities for being aware of others' intentions and forming participatory intentions to, say, write a paper together or play badminton necessarily would be compromised by this group of anomalous self-experiences.

To briefly summarize, this section sought to explain the aberrant social behavior in SSD by claiming that we-intentionality is fragile, whereas joint intentionality remains unaffected. Moreover, we have argued that group identification is a psychological precondition of we-intentionality and that group identification—and more specifically the interrelated process of self-categorization and de-individuation—can be destabilized by various anomalous self-experiences, which then render we-intentionality fragile. In this regard, disturbances of sociality can be seen as an integral part of the schizophrenia spectrum, as originally pinpointed by classical psychopathologists.

## Autism Spectrum Disorder

Before describing autism and assessing the functioning of shared intentionality in ASD, it is important to emphasize that our previous description of the two forms of shared intentionality and their psychological preconditions was framed from a developmentally advanced perspective. Turning now to developmental psychology and psychopathology, and more specifically to the case of autism in young children and toddlers, one should bear in mind that, as Hobson repeatedly has stressed (104, 105), what appears to be, from a developmentally advanced perspective, relatively distinct capacities (e.g., thinking, feeling, and willing) may *not* be clearly distinct capacities in infancy and early childhood. Moreover, these very capacities may *themselves* be achieved and relatively separated from each other on the basis of a complex social-emotional developmental process.

This observation also pertains to our own account of shared intentionality: some of the psychological preconditions, which we have described, are of course not available capacities in infancy and early childhood [see (27)]. Rather, they emerge fairly late in psychological development and thus arguably hinge on other, more basic factors. This is why, when exploring shared intentionality in children with severe ASD, we will not, as in the case of schizophrenia, assume the psychological preconditions are available and then explore ways in which they may be affected. Rather, we will key in on certain fundamental issues that seem to impede the emergence of the psychological preconditions for joint and we-intentionality in severe autism.

We now turn to how autism is defined in the diagnostic manuals. DSM-5 and ICD-10 concur in describing autism as a pervasive developmental disorder, which is characterized by deficits in social communication, in social interaction across multiple contexts (verbal, emotional, etc.), in restricted and often repetitive behavior, and in a limited range of interests. While ICD-10 (60) distinguishes between infantile autism (F84), atypical autism (F84.1), and Asperger's syndrome (F84.5), DSM-5 has replaced the diagnoses of autistic disorder and Asperger's disorder from DSM-IV-TR (106) with the diagnosis of ASD, and a similar nosological change will occur in ICD-11. ICD-10 states that some deficits in the above-mentioned domains of development are manifest before 36 months for infantile autism [(60), p. 253; (107), p. 147], and DSM-5 states that symptoms of ASD typically are recognized between 12 and 24 months [(61), p. 55]. Asperger's syndrome is defined by “the same kind of qualitative abnormalities of reciprocal social interaction that typify autism,” together with limited interests and restricted behaviors, but without clinically significant delays in cognitive development or retardation of language [(60), p. 258f; cf. (106), p. 80]. In the following, we will focus on social behavior in the severe end of ASD and explore it from the perspective of shared intentionality.

### Social Behavior in Severe Autism Spectrum Disorder

Aberrant social behavior has always been considered as a hallmark of autism. The current diagnostic criteria reflect some aspects of this social behavior, but they also, inevitably, ignore other aspects and qualities of such behavior. A few clinical examples, offered in the foundational texts on infantile autism by Kanner (73) and Asperger (74), may serve to illustrate characteristic forms of disturbed sociality in autism and help us key in on some of the central features. Despite the fact that almost 70 years has passed since the publication of these foundational texts, their clinical observations remain valid for infantile autism—even though they do not apply to what is nowadays defined as “Asperger's syndrome,” “high-functioning autism,” or the full spectrum of ASD. This gives us an opportunity to reinforce that our account aims at covering *severe* forms of autism, i.e., infantile autism, but does not apply to milder form of ASD. Our approach to aberrant social behavior in severe ASD further draws on the existing literature [e.g., (104, 105, 108)], and it supplements this extensive body of knowledge by addressing the topic from the perspective of shared intentionality.

In his original study, Kanner (73) described the case of a 4.5-year-old boy, Charles N., whose mother expressed her chief complaint as follows, “The thing that upsets me the most is that I can't reach my baby” [(73), p. 235]. She described her child as detached and as living “in a world of his own where he cannot be reached. No sense of relationships to persons.” She also said, “When he is with other people, he doesn't look up at them. Last July, we had a group of people. When Charles came in, it was just like a foal who'd been let out of an enclosure. He did not pay attention to them but their presence was felt (…) At school, he never envelops himself in a group, he is detached from the rest of the children, except when he is in the assembly; if there is music, he will go to the front row and sing” [(73), p. 236]. Charles N. displayed many of the signs that came to define the concept of autism such as repetitive behaviors or stereotypies (e.g., spinning toys for hours), preferring aloneness, avoiding eye contact, abnormalities of communicative exchange (e.g., echolalia, not responding to his own name, and reversing personal pronouns), restricted interests, and insistence on sameness in his routines.

These characteristic autistic features made the interpersonal relation between Charles and his mother very difficult, leading her to describe him as “unreachable” and “inaccessible.” In his concluding remarks, Kanner keyed in on this specific aspect of autism: “The outstanding, ‘pathognomonic,' fundamental disorder is the children's *inability to relate themselves* in the ordinary way to people and situations from the beginning of life” [(73), p. 242]. He further stated: “The children's *relation to people* is altogether different” [(73), p. 246], exemplifying it with (i) avoiding eye contact; (ii) not paying attention to other people present; (iii) not clearly registering persons coming and going; (iv) if an adult intruded in the child's game by hindering access to a desired object, the child would struggle with the obstructing hand or foot as a detached object, but would not attend the person, whose hand or foot it was; and (v) for the 6- to 8-year-olds, not playing *with* other children or participating in groups (though sometimes playing in the periphery of a group *alongside* other children), etc. [(73), p. 246–250]. Finally, Kanner famously concluded, “We must, then, assume that these children have come into the world with innate inability to form the usual, biologically provided affective contact with people, just as other children come into the world with innate physical or intellectual hand[i]caps” [(73), p. 250]. Notably, Kanner also emphasized some of the children's remarkable memory and good vocabulary.

The following year, Asperger published his study on “autistic psychopathy” in children (1944/1991), which bore strong resemblances to Kanner's study. Asperger also described autistic children's marked difficulties in social interaction, avoidance of eye contact, inability to play with other children or participate in groups, obsessive-like behaviors (close to what Kanner called “insistence on sameness”), hypersensitivity to sensuous stimuli, motorically clumsiness, stereotypic activities, and positive aspects of “autistic intelligence.” Asperger suggested that autism can occur at all levels of intellectual ability [(109), p. 58f, 74], and he argued that autistic features were visible early in development and temporally persistent: “From the second year of life we find already the characteristic features which remain unmistakably and constant throughout the whole lifespan” [(109), p. 67]^14^. He concluded, “the essential abnormality in autism is a disturbance in the lively relationship with the whole environment,” and this disturbance “explains all peculiarities shown by autistic individuals” [(109), p. 74]. A few pages later, he specified his claim as follows: “It has been my aim to show that the fundamental disorder of autistic individuals is the limitation of their social relationships” [(109), p. 77], and he argued that a “distinctive emotional defect” may be “an ultimate cause for their social disturbance” [(109), p. 80], which he then described as “a genuine defect in their understanding of the other person” [(109), p. 81].

Many of the clinical observations in these foundational texts have since been empirically corroborated and extensively elaborated. An important point, which is mostly implicit in these texts, however, is that children with severe ASD are not without communicative interests, though they communicate and interact less and differently than children without ASD or even with milder forms of ASD. They are also not insensitive to or unaffected by the presence of others (112). Furthermore, studies have dismissed the idea that autistic children and their mothers, despite the distress, cannot form secure attachments [e.g., (113, 114)], which seemed to be implied in Kanner's case of Charles N.

However, to sum up, following Kanner's and Asperger's insights, the essential problem in severe autism concerns *relating* to or *understanding* other persons *as* persons.^15^ As one autistic adult put it:

I really didn't know there were other people until I was seven years old … I then suddenly realized that there were people. But not like you do. I still have to remind myself that there are people … I never could have a friend. I really don't know what to do with other people, really [(116), p. 388; cited in (104), p. 3].

A 22-year-old autistic individual (Tony W.) who had been diagnosed with infantile autism nearly two decades prior offered the following description (text as in original):

I dont or didnt trust anybody but my self – that still (is) a problem today. And (I) was and still (am) verry insucure! I was very cold Harted too. I(t) was impossible for me to Give and Receive love from anybody. I often Repulse it by turning people off. Thats is still a problem today and relating to other people. I liked things over people and didnt care about People at all (…) My problems havn't changed at ALL from early childhood [(117), p. 50, 52].

Apart from highlighting forms of sociality that may appear strange for people without autism (e.g., the *realizing* that there are others, having to *remind* oneself that there are people or *turning people off* ), the descriptions also illustrate the autistic individuals' partial awareness of their own difficulties in relating to other persons.

### Joint Intentionality in Severe Autism Spectrum Disorder

Let us start with an investigation into the relation between severe ASD and joint intentionality. The first precondition for joint intentionality concerns mind reading abilities, which enable the subjects to become aware of the other agents' intentions and also to establish common knowledge among them about the fulfillment of the various requirements (where common knowledge is generally understood as a set of recursive beliefs ranging over others' beliefs about one's beliefs, etc.). The second precondition is that the subject should “be moved” by the other's intention in the sense of being willing to consider it and factor it in in her own deliberation and action planning. Remember that, in joint intentionality, the individual decides to act together with the other “partly because” of but also in “accordance with” the other's intention (24). Are these psychological preconditions met in severe ASD?

Let us now have a look at the first precondition and, in particular, with the capacity of tracking other agents' intentions. There is consensus in the literature that children with ASD are able to understand goal-directed actions [(118), p. 63, (105, 119)]. What remains a matter of debate is whether these patients are fully able to ascribe conative attitudes like intentions to others. First, it is controversial whether understanding goal-directed actions in young children amounts to understanding that a certain behavior is steered by a certain mental *attitude* (120, 121). Second, even granting the first point, it is unclear whether the kind of conative attitudes that children (with or without ASD) are able to understand is that of *intention*—in contradistinction to other kinds of conative states like wishes or desires^16^.

Regardless of how these issues will be settled, experimental studies have long demonstrated that children with severe autism show deficits in theory of mind and, therefore, have problems in forming beliefs about others' mental states (which, by extension, implies problems in establishing common knowledge with others). In a seminal study, Baron-Cohen et al. (108) demonstrated that children with autism have difficulties discriminating another's (false) belief about a situation from their own (correct) belief about it. Using Wimmer and Perner's design (125), Baron-Cohen, Leslie, and Frith introduced two doll protagonists, Sally and Anne. Sally had a basket and Anne had a box. Sally then placed a marble in her basket and left the scene. Anne now took the marble out of Sally's basket and hid the marble in her own box. Sally then entered the scene, and the child was asked the critical question “where will Sally look for her marble?” The authors examined children with infantile autism, children with Down's syndrome, and normally developing children; and they found that children with Down's syndrome and normally developing children scored similarly, where 86% and 85%, respectively, passed the test. By contrast, 80% of the children with autism failed the test—they all “incorrectly” pointed to the actual position of the marble. According to the authors, the children with infantile autism did not appreciate the difference between their own knowledge of the event and the knowledge that could be attributed to the doll [(108), p. 43]. Since the children with Down's syndrome, who had lower intellectual ability than the children with autism, performed well on test, failing the test could not be explained as a mere sequela of intellectual disability.

Interestingly, the authors described what they were testing as a “*conceptual* perspective-taking skill,” contrasting it with more traditional testing of “*perceptual* perspective-taking” such as “line of sight” or “three mountains” (where the child is confronted with the task of telling what can be seen from another, visual point of view [(108), p. 43f.]). Such tasks, they argue, may be solved solely by using visuo-spatial skills [e.g., (126)] and thus do not require attributing mental states to others. Finally, the authors refer to a study by Hobson (127), demonstrating that children with severe autism were no more impaired in *perceptual* perspective-taking tasks with doll protagonists than normally developing children matched on intellectual ability. Baron-Cohen, Leslie, and Frith conclude that the identified problem in the conceptual perspective-taking skill constitutes a specific cognitive deficit in ASD. In the following, we return to and dig deeper into this critical issue of conceptual perspective-taking in infantile autism.

For now, it suffices to state that literature on theory of mind, which covers more features than attributing false beliefs to others, show that individuals with severe autism typically have theory of mind deficits (128) and are impaired in the intuitive understanding that other people have mental states [(129), p. 283], or, as we put it earlier, in understanding persons *as* persons. This is sufficient for us to make the claim that individuals with severe ASD are likely to have difficulties fulfilling the first psychological precondition for joint intentionality. They encounter problems in tracking other intentions and in forming the recursive beliefs required for common knowledge.

What about the second precondition, i.e., the disposition to consider the other's intention and factor it in in the right way in one's conduct? In this respect, an important study by Hobson and Lee (130) has unveiled the difficulties for children with severe ASD precisely to be moved “according to” another's attitude. The study compared the way in which children with and without ASD acted after observing non-symbolic and non-conventional goal-directed actions performed by the experimenter by adopting different (and often idiosyncratic) styles of actions. Interestingly, children without ASD attempted to achieve the goal precisely by adopting the style or mode of action of the experimenter, i.e., by selective imitation^17^. According to the experimenters, this shows that the children without ASD were able to register and assimilate “another person's bodily anchored psychological stance (whether in feeling or action or some other way of relating to the world), in such a way that the stance becomes a potential way of the observer relating to the world from his or her own position” [(131), p. 411]. By contrast, children with ASD

were not moved to adopt the orientation of the person they were watching. They did not adopt the style with which the experimenter executed the actions, […] they were perfectly able to perceive and copy the strategies by which he achieved the goals in each demonstration. So they were able to learn something from watching what the experimenter did. They were also motivated to use what they had learned when their own turn came round. Yet what they learned seemed to be available from their position as a kind of detached observer of actions and goals. They were not “moved” [(132), p. 200].

Hobson's conclusion is reminiscent of Asperger's observation that children with autism have “an inability to learn from adults in conventional ways. Instead, the autistic individual needs to create everything out of his own thought and experience” [(109), p. 56]. It is crucial here to note that Hobson is using the expression “being moved” in a developmentally more primary sense than we have done so far. In our conceptual framework, “being moved” refers strictly to “being moved by the other's *intention*.” By contrast, what Hobson is arguing here is that children with infantile autism have a *relative* decreased propensity to identify with others' bodily anchored attitudes toward objects or events in the world, whereby children are rarely emotionally drawn or “moved” to assume the others' psychological attitude and, eventually, to acquire it as a potential attitude for themselves [(104, 105), p. 14–28, 131–140]^18^. It is plausible to conjecture that it is precisely because children with autism have a relative decreased propensity to identify with others that they also have difficulties factoring in the other's intentions when deliberating on how to pursue their own goal. Since the children rarely are “moved” in Hobson's sense of the term, they are also seldomly “moved” in the other sense that applies to the formation of participatory intentions.

If these observations are correct, then they indicate that individuals with severe ASD have problems with fulfilling both psychological preconditions of joint intentionality^19^. First, they have difficulties in tracking other intentions and establishing common knowledge with others. Second, they also have difficulties in forming participatory intentions “partly because” of and in “accordance with” the other's intention.

Before proceeding to the next section on we-intentionality in ASD, we would like to tackle a potential objection concerning our claims about the relation of joint intentionality and ASD, on the one hand, and joint intentionality and SSD, on the other. The reservation is this: the alleged difference we draw between the two disorders vis-à-vis joint intentionality (which has been claimed to be problematic in ASD, but unproblematic in SSD) is unsubstantiated because the same problems with theory of mind observed in ASD, and detrimental to joint intentionality, can also be observed in SSD. And this should indicate the existence of problems with joint intentionality in SSD, too (contrary to our claim).

As a reply to this objection, we offer the following considerations. As noted earlier, our account targets only severe ASD. We are aware of some findings from literature on milder forms of ASD, e.g., ascertaining that theory of mind deficits do not generally apply to persons with high-functioning autism (134). Other studies, comparing schizophrenia and ASD, have reported fairly similar theory of mind deficits in the two syndromes [e.g., (9, 135, 136)]. However, two observations are here in order. First, the empirical study by Pinkham et al. (9) and the majority of studies, examined in the meta-analysis by Chung et al. (135) and in the review and meta-analysis by Bliksted et al. (136), only included persons with ASD with an IQ > 70. Thus, as clearly pointed out by both Chung et al. [(135), p. 611] and Bliksted et al. [(136), p. 25), their findings are not generalizable to more severe ASD or to persons with severe ASD and intellectual disability. Second, the finding of comparable theory of mind deficits in schizophrenia and ASD also reflects the applied theory of mind tests. Notably, Doody et al. (137) applied the Sally-Anne test (a so-called first-order theory of mind test) to different patient groups, including schizophrenia. Not a single patient with schizophrenia (*n* = 28) failed the Sally-Anne test. Some problems were, however, observed in an additional second-order theory of mind test in patients with schizophrenia as well as in other diagnostic groups. This finding is echoed in a conclusion of a review on theory of mind deficits in schizophrenia, which states that understanding of first-order theory of mind problems is relatively preserved in schizophrenia (138). In our paper, we focus solely on infantile autism, and thus, given the observations above, the findings of comparable theory of mind deficits in high-functioning ASD (with IQ > 70) and SSD do not contradict our conclusions that joint intentionality is impaired in severe ASD but not in SSD.

### We-Intentionality in Severe Autism Spectrum Disorder

**Figure 1:**
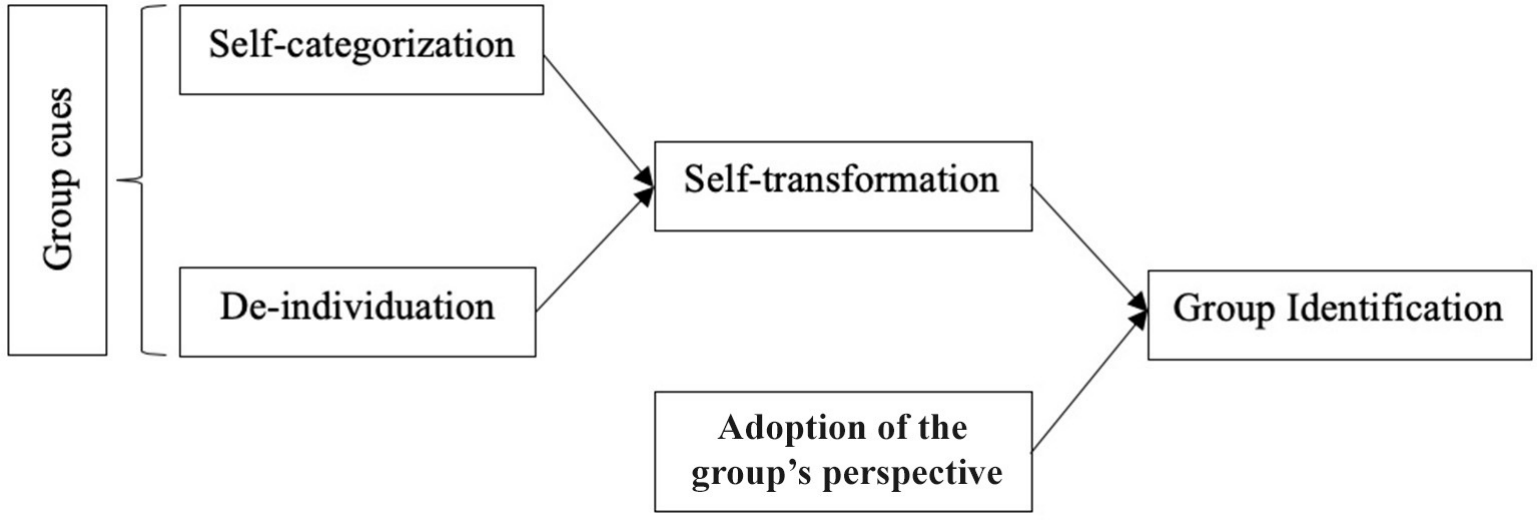
Group identification.

Turning now our attention to we-intentionality, the analysis will mainly focus on certain characteristic difficulties that seem to impede the emergence of the two psychological preconditions that enable group identification and thus this form of shared intentionality. These are the capacity to understand oneself as a group member (i.e., “transformation in self-experience”) and the ability to adopt the group's perspective (or we-perspective). Earlier, we have argued that the process of transformation in self-experience is initiated by the perception of group cues in the environment, which then triggers self-categorization and de-individuation (see Figure 1). Our discussion starts with the limited efficacy that group cues have in triggering group identification in subjects with ASD. We then move to transformation in self-experience, where we assess major difficulties that counteract especially self-categorization. We end with some speculative thoughts on why the adoption of the group perspective is impaired in the disorder.

As we have seen, some of the cues identified in social psychology research include sharing common interests or a common fate, facing a competing group, and using we-language. However, not all cues are equiprimordial from a developmental perspective, and one might doubt whether children of very young age are able to encode the properties at the basis of these cues. Yet at the same time, research into the early development of joint action has convincingly shown that children from the age of 18 to 24 months can engage in joint actions (139–142). Given the cognitive demandingness of joint intentionality (34, 143), it has been suggested that the joint actions in 18–24 months young children most likely are steered by some form of we-intentionality [see (27, 49)]^20^.

So what can facilitate we-intentionality in children of 18–24 months of age? One proposition that has been put forward [see (27, 34)] is that triadic joint attention may well play the required role here. To participate in an episode of triadic joint attention may sustain self-categorization because it is integral to the qualitative or phenomenal character of joint attention (i.e., to how joint attention is lived through by the subjects) that the participants see themselves as *co-attenders* and, arguably, that they are aware of sharing certain salient similarities. At this early stage of development, attending to the same object and perhaps with the same attitude, e.g., curiosity, is a sufficient salient similarity.

If triadic joint attention is relevant to we-intentionality, then difficulties with we-intentionality should be also expected in severe ASD, given that impairment in joint attention is a robust and predictively powerful indicator of severe ASD in young children (149)—as Trevarthen and his colleagues put it with regard to joint attention, here “autistic children appear characteristically impaired” [(115), p. 123]^21^. Accordingly, one group cue, which is particularly important from a developmental perspective, seems to be ineffective in triggering group identification in ASD.

But why is the ability to jointly attend to something problematic in ASD, and what evidence is there to support the claim that group identification and, specifically, transformation in self-experience are impaired in severe ASD? These questions are not unrelated, and, to answer them, we turn to Hobson's account of triadic joint attention, which he subsumes under the heading of “the relatedness triangle” (104). According to Hobson, triadic joint attention is not just a matter of two individuals, e.g., a child and a caregiver, attending to the same object in the world. In addition to relating to the object, the child also relates (i) to the caregiver, who reciprocally relates to the child, and (ii) to the caregiver's bodily expressed attitude or perspective on the object in the world. By socially and emotionally relating to the caregiver's bodily anchored and expressed attitude or perspective on an object in the world (e.g., a caregiver's curiosity toward a new toy), the child's own attitude or perspective on the object is potentially shaped or modified (e.g., the child's attitude may switch from feelings of uncertainty to curiosity toward the new toy).

More specifically, a significant developmental process is instigated when the child “moves to the position of the other,” thereby assimilating or assuming the bodily expressed attitude of the other and acquiring it as a potential attitude for itself [(104, 105), p. 14–28, 131–140]. As already noted, Hobson designates the crux of this developmental process with the concept of “identifying-with.” Most importantly, he distinguishes between different levels of identifying-with [(105), p. 17, 135], which roughly may be divided into two: first, a superficial form of identifying-with the other, enabling one to imitate or copy the other's goal-directed behavior; and second, a deeper form of identifying-with the other in which one is emotionally drawn or “moved” to assume the other's bodily anchored psychological attitude or perspective, enabling one's own attitude or perspective to be configured according to what is perceived in the other (e.g., the beforementioned shift from uncertainty to curiosity toward a new toy). According to Hobson, it is pivotal for the emerging social understanding that the infant “registers this shift *as* a shift across perspectives, not merely as a change in the meaning of objects at the focus of referencing” [(105), p. 137]. In other words, the deeper form of identifying-with is quintessentially person-centered [(105), p. 138], which means that the infant experiences the shift in her own attitude toward the object or event as mediated by another person. In this process, the child is “lifted out of her own stance and (…) drawn into adopting another perspective” [(151), p. 106, 108]. By repeatedly engaging in triadic joint attention and by shifting between self/other perspectives based on the deeper form of identifying-with, the child gradually comes to understand not only that there are different perspectives on the same objects and that she herself can be an object of another's perspective but also, eventually, that persons are sources of perspectives and that she herself is a person with a perspective [(105), p. 106]. Leaving other details of Hobson's account aside, one can conclude that, on that view, triadic joint attention, based on the deeper form of identifying-with the other, is crucial for coming to understand others *as* persons as well as oneself *as* a person.

Where does this leave us in the case of severe ASD? According to Hobson, children with infantile autism manifest a “negative image” of triadic joint attention (or the relatedness triangle)—as he puts it, “It is especially when a normal child would be attending to, registering, evaluating, and identifying with the *subjective orientation* of another person, that the autistic child is the most abnormal” [(104), p. 197]. Said another way, children with severe ASD are typically not impaired when it comes to the superficial form of identifying-with the other. However, when it comes to the developmentally crucial, deeper form of identifying-with others, children with severe ASD are markedly impaired [(105), p. 14–28, 131–140]. On Hobson's account, this decreased propensity to identify with others—this relative impairment in the capacity to be “emotionally moved” to assume the other's subjective attitude or perspective and acquire it as a potential perspective for oneself—is *the* generative disorder in infantile autism or, as he also puts it, “what makes autism autism” [(105), p. 131].

This fundamental disorder reverberates in other aspects of sociality: it is well-known that infants with severe ASD regularly not raise their arms to be picked up, have decreased eye contact, have impoverished proto-declarative pointing^22^, have a failing grasp of others' use of proto-declarative pointing, often do not participate in turn-taking with adults, and rarely show objects to others, etc. Their social impairments are also mirrored later in life. In a series of experimental studies of social emotions, Hobson et al. (105) found important group differences between children and adolescents with severe autism, and children and adolescents with developmental delays and learning disabilities (without autism). For example, children and adolescents with severe autism were less likely to manifest person-focused social emotions such as shame and guilt and their manifestation of these emotions were atypical—e.g., they rarely reported feeling guilty for hurting someone but rather guilty for breaking a rule. Furthermore, children and adolescents with severe autism frequently described and expressed pride but, again, in an atypical, non-person-focused manner than the developmentally delayed control group—children and adolescents with autism expressed pride over their own achievements but appeared indifferent when praised for their achievements by others. In another study, Lee and Hobson (153) examined self-concepts in adolescents with autism and a matched control group with intellectual disability and found notable group differences with regard to social self-statements in terms of *quantity* (adolescents with autism produced less social self-statements, e.g., about helping others or being bullied) and *quality* [not a single adolescent with autism referred to a friend (whereas 70% of those without autism did) or to being a member of a social group]. In brief, children with severe ASD have basic problems in relating to others—problems that cannot be explained merely by intellectual disability—and these problems predate and most likely also constrain and structure the development of a range of other capacities, including social emotions and what Baron-Cohen and colleagues called “conceptual perspective-taking.”

Returning now to the psychological preconditions of group identification, we suggest that the fundamental problems involved in conceptual perspective-taking in severe ASD hampers these very preconditions of we-intentionality. We first look into difficulties related to self-transformation and, specifically, self-categorization before turning our attention to the adoption of the group's perspective.

To start with self-categorization, this process, as we saw, leads to a self-perception as an individual saliently similar to others. It thus presupposes the possibility to relate to oneself in a specific way which, importantly, takes others into consideration. I should have a, however rudimentary, sense of myself but also of others as minded beings (like me) to become aware of significant similarities between us (154). If impairments in triadic joint attention, grounded in a decreased propensity for deeply identifying-with others and subsequent problems in conceptual perspective-taking, etc., entail fundamental problems in relating to others and oneself *as* persons, then these very same problems will also affect the process of self-categorization and, consequently, of self-transformation. Our interim conclusion, thus, is that self-transformation is impaired in ASD.

Finally, the last precondition for we-intentionality is the ability to adopt the group's perspective. Philosophical research has argued that adopting the group's perspective could be described as a form of perspective-taking (27, 155). In the adoption of the group's perspective, just as in other forms of perspective-taking, the subject adopts the perspective of another agent; it just is that, here, the perspective that the agent adopts is the perspective of a group agent and not that of another individual. Although, to the best of our knowledge, there are no empirical studies yet to support this conjecture, it seems reasonable to suggest that, if children with severe ASD have fundamental problems with adopting the perspective of others (conceptual perspective-taking), then those very same problems with perspective-taking may also affect the capacity to adopt the group's perspective and to factor it in in deliberation and action planning.

We conclude that both joint intentionality and we-intentionality are impaired in severe ASD, since the psychological preconditions of these forms of shared intentionality appear not to be met.

## Conclusions

We have proposed that shared intentionality comes in at least two different forms, namely, joint intentionality and we-intentionality, and we have suggested that these two forms require different psychological preconditions to be established and maintained. In joint intentionality, the agents' motivation and perspective are *individual*, and for them to lead to joint action, they must be accompanied by robust mentalizing abilities. By contrast, in we-intentionality, the agents act on collective motivation and perspective, as they must be able to adopt the group perspective and act in accordance with the group's preferences and goals.

With regard to joint intentionality, we have argued that it is impaired in severe ASD but not in SSD and that the impairment in severe ASD may be caused by problems with mind reading and with the ability to “be moved” by others' intentions. With regard to we-intentionality, we have argued that the presence of various, trait-like anomalous self-experiences may exert friction on the psychological preconditions for self-transformation and thus render we-intentionality fragile in SSD. In severe ASD, by contrast, we have argued that fundamental problems involved in perspective-taking seem to violate the psychological preconditions for group identification and thus we-intentionality. Although we-intentionality appears to be affected in both SSD and ASD, the root problems are different, linked to the disorders' specific psychopathological cores, and result in qualitatively distinct difficulties in this domain of social interaction.

Our analysis of disturbed shared intentionality in SSD and ASD also made it clear that the psychological preconditions for joint intentionality and we-intentionality, which we described from a developmentally advanced perspective (see Table 2), are, in fact, not able to fully account for these two forms of shared intentionality. In these analyses, it became evident that for these psychological preconditions to work, other and developmentally more primary factors need to be in place. For example, for group cues to bring about self-transformation and, eventually, group identification, a certain sense of oneself as a *person*, of others as *persons*, of groups as consisting of *persons*, and not least the basic capacity to be “emotionally moved” by others, is indeed required.

Finally, it of course merits attention that the hypotheses that we have put forth here concerning disturbances of these two forms of shared intentionality require empirical corroboration before any definitive conclusions can be drawn on these complex matters.

## Data Availability Statement

The original contributions presented in the study are included in the article, further inquiries can be directed to the corresponding author.

## Author Contributions

All authors listed have made an equal, substantial, direct and intellectual contribution to the work, and approved it for publication.

## Conflict of Interest

The authors declare that the research was conducted in the absence of any commercial or financial relationships that could be construed as a potential conflict of interest.

## Footnotes

^1^Two comments are in order. The first is that, although closely related, social cognition and shared intentionality are conceptually distinct capacities: whereas the first capacity is about understanding others' mental states, the second capacity (esp. when it narrowly refers to shared *intentions qua* conative states, see the next section) concerns the *motivation* to engage in pro-social behavior and collaborative actions with others (12). The debate on shared intentions has been sparked almost 30 years ago by the insight that the second capacity does not boil down to the first [see (1)]. The second comment is that, of course, we do not mean to imply that other psychiatric disorders (e.g., organic or affective psychosis, personality disorders, or social anxiety) are not confronted with problems of sociality and that an investigation into shared intentionality could not shed light to these disorders, too [see (13)].

^2^Although the current paper builds upon Salice and Henriksen (10), it also substantially advances and, in certain cases, rectifies the view we develop there. In addition to including infantile autism in the account, the current paper offers a more precise analysis of shared intentionality. As the next sections show, this analysis relies on a refined understanding of group identification (as an umbrella term that encompasses two different processes: self-transformation and the adoption of the group's perspective) and on an equally refined understanding of what it means to factor in another's intention (or “to be moved by” that intention) in deliberation. Also, the current analysis does not any longer hinge on a taxonomy of groups.

^3^Note that we do not argue that the factors, which we discuss in this paper, are the only ones that may affect shared intentionality in ASD and SSD. For example, based on the idea that awareness of others' mental states does not rely only on mentalizing capacities but is also related to intersubjectively constituted embodied attunement, Fuchs (11) has argued that a disorder at the level of “pre-reflective embodied relationship of self and other” may be a cause of disruption for intersubjectivity in the two syndromes.

^4^On shared beliefs, see the classical work by Gilbert (18). For recent work on shared emotions, see von Scheve and Salmela (19).

^5^Perhaps not surprisingly, not everybody agrees on this point. For instance, Searle (22) and Wilby (37) argue that for individual intentions to steer a joint action, they must be held by the agents from the group's perspective (they are “we-intentions”). Despite substantial differences, the account of we-intentionality, which we develop in *We-Intentionality* section, broadly aligns with this approach. However, one bone of contention is whether we-intentionality is the *only* psychological power that can steer a joint action, and this is what we deny (as this section on joint intentionality illustrates).

^6^This seems clear enough in large groups, but what about in small dyads? Would not a change of mind in one of the two parties necessitate a change of mind in the other and, therefore, the collapse of the joint activity? In actual practice, this might well be the case, but this possibility still remains contingent on the following, namely, that members are licensed to do so only under the understanding that both of them have reneged on the commitment toward their goal. Often, that understanding remains tacit, but this is simply because the members typically know each other well and consider this procedure for dissolving the commitment permissible [see on this in (38)].

^7^Note that this does not exclude the possibility for individuals to contribute to a group's goal for other reasons as well (e.g., conformism and reputation management).

^8^One behavioral outcome of the adoption of the group's perspective is the so-called “black sheep effect,” which can be detected already at the age of three (46) but manifests itself in its mature form from the age of eight (47). This effect is evident in the way in which loyalty or disloyalty to a group is assessed: loyal behavior of in-group members is praised more than a similar behavior by out-group members; and deviant, or disloyal, behavior of in-group members is punished more severely than a similar behavior by out-group members. Importantly, since the tokens of behaviors that are assessed do not differ in their properties, there is nothing that makes these tokens of behaviors intrinsically better or worse. This illustrates that, when the perspective of the group is factored in, the assessment of the behavior in question diverges: a loyal action toward group G is assessed as more praiseworthy than loyal actions toward other groups only from G's perspective. Conversely, it is only from G's perspective that a deviant action toward G is punished more harshly than deviant actions toward other groups.

^9^On this note, it might be important to remark that we-intentionality has the disposition to sediment and to solidify through time, enabling the existence of large groups animated by a sense of cohesion that is sustained by a shared social identity (3, 51).

^10^By “common sense,” we understand the body of “hinges propositions” (54) that enable our (individual or collective) agency in a shared world. These are propositions that “stand fast” for the agents and deliver their “primitive certainties.” Accordingly, Wittgenstein's hinge propositions are to some extent similar to Searle's “background capacities” [(55), p. 175–96], which are not really beliefs but rather ways of behaving that manifest that something has been taken for granted [(56), p. 112–13]. In the psychiatric literature, Blankenburg (57, 58) has offered a detailed account of loss of common sense as a central feature of schizophrenia.

^11^Although it exceeds the purposes of this paper to elaborate on this, it merits attention that one of us has argued that we-intentionality developmentally precedes joint intentionality [see (27, 28, 38, 59); for a similar view, see (41)].

^12^We write “predominantly” because the kind of activity at stake *per se* is not revelatory of the kind of shared intentionality that steers it. One and the same activity (e.g., karate, writing a paper, or cooking dinner) can be engaged in by joint intentionality or we-intentionality. What matters is how the agents frame the activity and, in particular, whether the activity's goal is shared distributively or collectively. We come back to this point below.

^13^What sort of representation is the social self, viz., this peculiar understanding of oneself as a group member? In related work (27), we have argued that this is neither a doxastic state (like beliefs and perceptions) nor a conative state (like intentions and desires). Understanding oneself as a group member, in the sense at stake here, is to be in a state that at once describes the subject as a group member and motivates her to act as such. In the literature, different authors have labeled states as these differently: “Pushmi-Pullyu Representations” (82), “Aliefs” (83), or “Interested Participatory Representations” (84).

^14^Asperger claimed that social adaption and integration in adulthood are to some extent possible but depend especially on the individual's intelligence. Concerning the differential–diagnostic boundaries between autism and schizophrenia, which today has become a topic of debate [e.g., (88, 110, 111)], Asperger, like Kanner, argued that they were distinct conditions, and Asperger explicitly denied the possibility that childhood autism could be a precursor for schizophrenia [(109), p. 86; cf. (73)].

^15^Kanner's and Asperger's attempts to articulate the generative disorder of autism in terms of “disturbance of affective contact” or “emotional defect” have since been challenged, especially by cognitive or meta-cognitive accounts [e.g., (108)]. Hobson's account, as we shall see later, can also be viewed as challenging the “affective” accounts of Kanner and Asperger as well as the more “cognitive” accounts insofar as he locates the systemic disorder in a basic form of relatedness that is irreducibly cognitive/conative/affective in nature [(105), p. 7, 131f]. For a brief overview of positions, see Trevarthen et al. [(115), p. 123–126].

^16^On the distinction between these conative states, see Bratman (122). On children's understanding of various kinds of conative states, see Astington (123) and Perner (124).

^17^For instance, the “rolling policeman” is “a 10 cm high plastic toy policeman that stood on wheels. When the policeman was pushed down from above, a spring mechanism operated so that the policeman moved forward across the table under his own steam. The two styles that [the experimenter] used in operating the rolling policeman were as follows. In one case [the experimenter] cocked back his right hand and depressed the head of the policeman with the front of his wrist: in the other case he extended his index and middle finger, and used these to press down on the head” [(130), p. 655]. Whereas children without autism largely imitated the styles of the experimenter after observing them, the vast majority of autistic children did not imitate properly by activating the mechanism in any of the two styles but just used the palm of their hand to press on the head.

^18^Hobson employs the notion of identification in a technical sense, which should not be confounded with the other technical concept of group identification we discussed above (to mark Hobson's specific notion, we hyphenate, as he does, the notion of “identifying-with”).

^19^Let us emphasize again that our considerations only apply to severe autism. In fact, it has been suggested that Asperger patients may be able to engage in activities steered by joint intentionality (133).

^20^One argument supporting this claim is that, in children, the ability to engage in joint action emerges earlier than the development of mindreading abilities. Passing the false belief test, e.g., the Sally-Anne test, at the age of 4 has traditionally been considered the first reliable mark of theory of mind abilities (125, 144, 145). While some theory of mind tests [so-called “spontaneous-response” (146) or “indirect” tests (147) in contrast to classical “elicited-response” tests like the Sally-Anne test] predate the emergence of these abilities to 2.5 years of age (148), if not even to 13 months of age (146), they still do not align with the developmental emergence of joint actions.

^21^This point will not apply to patients with high-functioning autism of whom it has been ascertained that they may point to social interaction as one of their favorite activities [e.g., see (150)].

^22^Goodhart and Baron-Cohen define proto-declarative pointing as “pointing to comment or remark on the world to another person, to share interest or attention about an object, as an end it itself” [(152), p. 226]. Proto-declarative pointing is distinguished from proto-imperative pointing, which is pointing “to use another person to obtain an object” (152).
